# North American *Culex pipiens* and *Culex quinquefasciatus* are competent vectors for Usutu virus

**DOI:** 10.1371/journal.pntd.0006732

**Published:** 2018-08-17

**Authors:** Christian L. Cook, Yan-Jang S. Huang, Amy C. Lyons, Barry W. Alto, Isik Unlu, Stephen Higgs, Dana L. Vanlandingham

**Affiliations:** 1 Department of Diagnostic Medicine/Pathobiology, College of Veterinary Medicine, Kansas State University, Manhattan, Kansas, United States of America; 2 Biosecurity Research Institute, Kansas State University, Manhattan, Kansas, United States of America; 3 Florida Medical Entomology Laboratory, University of Florida, Vero Beach, Florida, United States of America; 4 Mercer County Mosquito Control, West Trenton, New Jersey, United States of America; 5 Center for Vector Biology, Rutgers University, New Brunswick, New Jersey, United States of America; Center for Disease Control and Prevention, UNITED STATES

## Abstract

**Background:**

Usutu virus (USUV) is a member of the Japanese encephalitis virus (JEV) serocomplex in the *Flaviviridae* family. Emergence of USUV in Europe has led to disease burdens in birds and created increasing concern for the potential zoonotic transmission to humans. Whilst USUV has not been detected in the New World, the identification of competent vector species in North America is critical in the assessment of the likelihood of its dispersal and establishment of enzootic transmission cycles. The objective of this study was to determine vector competence of potential mosquito vectors in North America for USUV. Three medically important mosquito species were selected for testing because of their involvement in the transmission of West Nile virus and St. Louis encephalitis virus, two related JEV serocomplex flaviviruses in the New World.

**Methodology/Principal findings:**

Oral challenge of *Culex pipiens*, *Culex quinquefasciatus*, and *Aedes albopictus* was performed to determine the susceptibility and vector competence of North American mosquitoes for USUV. Infection status was monitored by the isolation of virus from homogenized mosquito tissues. The disseminated form of infection was demonstrated by the detection of infectious virus in the head, wings, and legs of infected mosquitoes. The presence of viral RNA in saliva of infected *Cx*. *pipiens* and *Cx*. *quinquefasciatus* indicated that both species are competent for transmission of USUV.

**Conclusions/Significance:**

Results indicate that members of the *Cx*. *pipiens* complex are susceptible to USUV and competent for its transmission potential in North America in the event of its introduction. In contrast, *Ae*. *albopictus* were highly refractory to USUV infection, suggesting that this species is unlikely to contribute to USUV transmission in North America.

## Author summary

Usutu virus is an emerging mosquito-borne flavivirus maintained between avian and mosquito species. Although the pathogen has only been detected in Africa and Europe, a growing concern of its dispersal and zoonotic potential warrants the investigation on the vector competence of mosquito species outside the endemic regions for USUV. Identification of species involved in the transmission cycles allows for the formulation of disease control strategies such as vector control. As observed with West Nile virus and Japanese encephalitis virus, the presence of competent vector species is critical for the establishment of enzootic transmission cycles and contributes to the viral maintenance in nature. Despite differences in the genetics and bionomics of *Culex pipiens* in the Old World, members of the *Cx*. *pipiens* species in the New World are competent for USUV based on the results of *per os* challenges performed in this study. *Cx*. *pipiens* and *Cx*. *quinquefasciatus* are capable of initiating enzootic transmission cycles in the presence of susceptible avian species and should be targeted for vector control in the event of a disease outbreak.

## Introduction

Usutu virus (USUV) is a mosquito-borne flavivirus classified under the Japanese encephalitis virus (JEV) serocomplex in the *Flaviviridae* family. Usutu virus was originally isolated in 1959 in South Africa [1]. The earliest dispersal of USUV from the African continent was through the detection of viral RNA in tissues of the common European blackbird, *Turdus merula*, in Italy in 1996 [2]. Since 2001, the emergence of USUV has been continuously reported throughout Europe. It has been reported in Belgium, Czech Republic, England, Hungary, Italy, Germany, Poland, Spain, and Switzerland; and has been responsible for the deaths of thousands of birds with an estimated 15.7% blackbird population decline attributed to the virus [3, 4]. It has also become a potential public health threat after reports of neurotropic diseases in humans.

Exposures to USUV typically lead to mild diseases and seroconversion in incidental hosts as observed in humans and horses [5]. Historically, human USUV infections have been primarily associated with self-limiting febrile illness and rash followed by the development of neutralizing antibodies. USUV infection leading to neurotropic disease was only documented in immunocompromised individuals [6, 7]. However, more recent evidence indicates that severe disease could also take place in healthy adults which has considerably increased the public health significance of USUV [8, 9].

Similar to other members of the JEV serocomplex, maintenance of USUV in nature depends on the enzootic cycle among viremic avian species as amplification hosts. Transmission of USUV in Africa has been demonstrated to be vectored by ornithophilic mosquito species such as *Culex neavei* [10] with additional isolates from pools of *Cx*. *pipiens*, *Cx*. *univitattus*, and *Coquillettidia aurites* [1, 11, 12]. Although isolates of USUV have been made from various zoophilic mosquitoes including *Cx*. *pipiens*, *Cx*. *modestus*, *Aedes albopictus*, *Ae*. *caspius*, and *Anopheles maculipennis* in Europe since 2001, vector competence studies suggest that USUV is mainly transmitted by competent *Cx*. *pipiens* populations [13–18].

As historically observed with the dispersal of multiple flaviviruses in the JEV serocomplex, enzootic transmission can be quickly established in the presence of competent vectors and amplification hosts. The emergence of USUV in Europe during the last two decades has significantly increased the likelihood of its further dispersal into other geographic regions including the United States. In this study, oral challenge with USUV was performed in North American *Cx*. *pipiens*, *Cx*. *quinquefasciatus*, and *Ae*. *albopictus* to determine their vector competence for USUV. The three species were selected based on their known involvement in the transmission of West Nile virus, a flavivirus within the JEV serocomplex, in North America.

## Materials and methods

### Cells and virus

African green monkey kidney epithelial Vero76 cells maintained in L-15 medium supplemented with 10% fetal bovine serum, 10% tryptose phosphate, penicillin, streptomycin, and L-glutamine were used in this study for propagation of virus stocks and titration of all infectious materials as previously described [19]. The prototype SAAr1776 strain was used for all *per os* infection of mosquitoes. The strain was originally isolated from a pool of *Cx*. *neavei* in South Africa in 1959. Prior to the experiment, the strain obtained from the Division of Vector-Borne Diseases, Centers for Disease Control was passaged seven times in suckling mouse brain and twice in Vero cells. Two additional passages in Vero76 cells were performed in our laboratory to generate stocks for *per os* challenge. Phylogenetically, the SAAr1776 strain forms a monophyletic cluster with recent isolates of USUV in Europe and has the closest relatedness to the sequence of USUV MB119/06 isolated in Spain in 2006 [20].

### Mosquitoes and *per os* infection

Colonies of *Cx*. *pipiens* (F_21_) and *Cx*. *quinquefasciatus* (F_21_) used in this study were established from larvae collected from Ewing Township, Mercer County, NJ and Vero Beach, FL in August 2015 as previously described [21]. The colony of *Ae*. *albopictus* (F_4_) tested in this study was derived from eggs collected from the City of Trenton, Mercer County, NJ in July 2016. All mosquitoes were maintained with 20% sucrose solution under a 16h: 8h light: dark photo regimen at 28°C. Eight to ten-day old mosquitoes were deprived of sugar and water 48 and 24 h before *per os* infection, respectively. USUV stock and defibrinated sheep blood (Colorado Serum Company, CO) were mixed in equal volumes to produce artificial viremic blood meals. Blood meals were administered to *Culex* species mosquitoes in gallon size cartons through cotton pledgets at room temperature for 1 h. Oral challenge of *Ae*. *albopictus* was performed with a Hemotek membrane feeding apparatus as previously described [22]. Control groups received defibrinated sheep blood mixed with tissue culture media. Ingestion of defibrinated sheep blood by control groups ensures that cytopathic effect in Vero76 cells used as an indicator for viral infection was caused by the infection of USUV and not by other pathogens present in mosquitoes. Titers of viremic blood meals were determined by titration of remaining blood meals aspirated from cotton pledgets and individual blood feeders after the completion of each *per os* infection experiment. Fully engorged mosquitoes were collected under cold anesthetization and returned to the cartons. Three engorged mosquitoes in each group were frozen immediately after receiving blood meals to confirm ingestion of USUV and to quantify the viral titer of the infected blood ingested by mosquitoes.

Mosquitoes were sampled at 7 and 14 days post-infection (d.p.i.) to characterize the infection process of USUV. To determine the incidence of disseminated infection, dissection of mosquitoes was performed to separate the abdomen from the head, wings, and legs. The abdomen contains the midgut where the infection of arboviruses is initially established. The head, wings, and legs are the secondary tissues, which are infected with viruses disseminated from the midgut. A second group of mosquitoes was collected, without dissection, to determine replication kinetics of USUV in whole mosquitoes. At 14 d.p.i., saliva of each mosquito was collected through forced salivation for 60 minutes prior to dissection, as previously described [19, 23]. Titers of viremic blood meals and whole mosquitoes sampled at 7 and 14 d.p.i. are summarized in Table 1.

**Table 1:** Summary of titers of viremic blood meals and whole-mosquito of usutu virus at 0, 7, and 14 d.p.i.

Mosquitoes Tested	Average Titer of Viremic Blood Meals	Average Whole-Mosquito Titer		
		0 d.p.i.	7 d.p.i.	14 d.p.i.
*Cx*. *pipiens*	7.52	3.31 ± 0.17	4.3±0.42	4.6±0.88
*Cx*. *quinquefasciatus*	6.95	4.14 ± 0.19	5.5±0.16	4.3±0.34
*Ae*. *albopictus*	5.95 ±0.58	2.95 ± 0.07	Undetected	Undetected

Titers listed in log_10_TCID_50_/mL

### Detection of USUV in mosquito tissues and saliva

To quantify infectious viruses, all samples containing mosquito tissues were homogenized using a TissueLyser II apparatus (Qiagen, MD) at 26 Hz for 4 min and titrated by 50% tissue culture infectious dose (TCID_50_) method with Vero76 cells as previously described [24]. The establishment of infection was defined by the detection of USUV in the body or secondary tissues from dissected mosquitoes or in homogenized whole mosquitoes. Infection rates were calculated based on the overall percentage of infected mosquitoes among mosquitoes tested. Viral dissemination was demonstrated by the detection of USUV in the secondary tissues of infected mosquitoes. Dissemination rates were derived from the incidence of positive detection of infectious viruses in secondary tissues among dissected mosquitoes infected with USUV.

A nested reverse transcriptase-polymerase chain reaction (RT-PCR) was used to detect the presence of USUV in the saliva of infected mosquitoes. The transmission rate of USUV was determined by the incidence of positive detection of viral RNA among infected mosquitoes. Viral RNA was extracted using the QIAamp Viral RNA Mini Kit (Qiagen, MD). Complementary DNA was produced by reverse transcription of Superscript III reverse transcriptase (Life Technologies, CA) with the VD8 primer that is broadly reactive to the 3’ untranslated region of all mosquito-borne flaviviruses [25]. The first amplicon was produced using Platinum *Taq* polymerase (Life Technologies, CA) using the VD8-EMF1 primer set as previously described [25]. The second amplicon was derived from the second RT-PCR reaction using two USUV-specific primers, USUV 10268–10287 (5’-GCAACGTGGGCTGAAAACAT-3’) and USUV 10821–10802 (5’-AGTTCGCATCACCGTCTGTT-3’). The USUV-specific primer set was designed using the Primer-BLAST algorithm available from the website of National Center of Biotechnology Information. All amplicons were separated and visualized by electrophoresis using 1% agarose gel.

### Statistical analysis

Infection, dissemination, and transmission rates of USUV in infected mosquitoes were analyzed using Fisher’s exact test. All statistical analyses were performed using SigmaPlot version 12.0 (Systat Software Inc., CA).

## Results

### Infectious viruses of USUV isolated from orally challenged *Cx*. *pipiens* and *Cx*. *quinquefasciatus*

As summarized in Table 1, USUV was detected in all three mosquito species immediately after engorgement. Engorgement of infectious viruses led to establishment of infection in both *Cx*. *pipiens* and *Cx*. *quinquefasciatus* (Table 2). Replication of USUV led to significant increases in the infection rates of *Cx*. *pipiens* from 25% (4/16) at 7 d.p.i. to 58.6% (17/29) at 14 d.p.i. (*p* = 0.03) and disseminated infection at 14 d.p.i. There was no significant difference in the infection rates of USUV in *Cx*. *quinquefasciatus* at 7 (93.3%, 14/15) and 14 (70.0%, 21/30) d.p.i. Similarly, the dissemination rate of USUV in *Cx*. *quinquefasciatus* was not significantly different at 7 (66.7%, 4/6) and 14 (35.7%, 5/14) d.p.i. Despite *Ae*. *albopictus* being challenged with similar titers of infectious virus as ingested by *Cx*. *pipiens*, *Ae*. *albopictus* were highly refractory to USUV infection, with no detection of virus at 7 (0.0%, 0/22) or 14 (0.0%, 0/27) d.p.i.

**Table 2:** Infection and dissemination rate of usutu virus at 7 and 14 d.p.i.

Mosquitoes Tested	Infection Rate		Dissemination Rate	
	7 d.p.i.	14 d.p.i.	7 d.p.i.	14 d.p.i.
*Cx*. *pipiens*	25% (4/16)	58.6% (17/29)	100%(1/1)	92.3% (12/13)
*Cx*. *quinquefasciatus*	93.3% (14/15)	70% (21/30)	66.7% (4/6)	35.7% (5/14)
*Ae*. *albopictus*	0.0% (0/22)	0.0% (0/27)	N/A	N/A

N/A = not applicable

### Detection of viral RNA in saliva of infected mosquitoes

The detection of infectious viruses in *Cx*. *pipiens* and *Cx*. *quinquefasciatus* indicated that both species are susceptible to USUV through oral exposure. To evaluate potential for transmission by both species, nested RT-PCR was used to detect viral RNA in the saliva. Among infected *Cx*. *pipiens*, 23.5% (4/17) of saliva samples collected at 14 d.p.i. were positive for the presence of USUV viral RNA. Similarly, 19.0% (4/21) of infected *Cx*. *quinquefasciatus* had positive detection of viral RNA in saliva at 14 d.p.i.

## Discussion

Our results demonstrate that North American *Cx*. *pipiens* and *Cx*. *quinquefasciatus* are susceptible to USUV and competent for its transmission. With the evidence demonstrating the vector competence of *Cx*. *pipiens* for USUV and its involvement of transmission in nature, the species is likely to be a critical player for the further dispersal of USUV in the future and a target species for vector control in the event of disease outbreak in North America.

Whilst there was no significant difference in infectious titers of whole mosquitoes collected at 0, 7, and 14 d.p.i., a minor increase in the infectious titer and the significant increase in infection rates observed in *Cx*. *pipiens* indicate that the species is permissive for the replication of USUV. Similarly, *Cx*. *quinquefasciatus* tested in this study also allowed the replication of USUV as demonstrated with maintenance of infectious titers of whole mosquitoes at 7 and 14 d.p.i. The replication of USUV in both species ultimately led to its dissemination from the midgut into secondary tissues including salivary glands, as demonstrated by the detection of viral RNA in the saliva of infected mosquitoes at 14 d.p.i.

Whilst *Ae*. *albopictus* have been demonstrated to be competent for the transmission of multiple flaviviruses in the JEV serocomplex and a source of naturally occurring isolates of USUV [26], the species has been demonstrated to be highly refractory to USUV based on our and others’ findings [18, 27]. As USUV is unable to establish infection in orally exposed *Ae*. *albopictus*, the species is unlikely to be involved in the transmission and maintenance of USUV. Isolation of infectious viruses or detection of viral RNA from *Ae*. *albopictus* may be a consequence of recent engorgement from viremic avian species as some *Ae*. *albopictus* populations have been demonstrated to have opportunistic feeding behaviors and utilize avian species as a source of blood meals [28].

Despite the known genetic differences between members of the *Cx*. *pipiens* complex in the Old and New World and differing methods for detection of USUV [29], *Cx*. *pipiens* and related species remain as the most probable vector for USUV in North America. Notwithstanding the variance in methods, our results are comparable with the previous publication on the vector competence of populations of *Cx*. *pipiens* in Northwestern Europe for USUV [17]. Our observations also do not exclude the possibility that other mosquito species can potentially serve as competent vectors for the transmission of USUV outside its endemic regions. As observed with JEV and WNV, dispersal of flaviviruses under the JEV serocomplex often involves transmission by multiple mosquito species in nature. This strategy may ultimately increase the likelihood for the permanent establishment of enzootic transmission cycles and challenge the efforts in the formulation of vector control strategies.

Whilst there have been at least two JEV-serocomplex members present in the New World including WNV and St. Louis encephalitis virus (SLEV), USUV remains likely to be established and co-circulate with related viruses in the potential event of its introduction. As co-circulation of USUV and WNV have been reported in Europe, it is highly unlikely that the potential introduction of USUV will lead to the displacement of WNV [26]. Whether or not co-circulation can ultimately result in the displacement of SLEV, as seen with the displacement of SLEV by WNV in California in 2003, remains unclear.
